# Band Offsets and Interfacial Properties of HfAlO Gate Dielectric Grown on InP by Atomic Layer Deposition

**DOI:** 10.1186/s11671-017-2104-y

**Published:** 2017-05-08

**Authors:** Lifeng Yang, Tao Wang, Ying Zou, Hong-Liang Lu

**Affiliations:** 1 Shanghai Synchrotron Radiation Facility, Shanghai Institute of Applied Physics, Chinese Academy of Sciences, Shanghai, 201800 China; 2 State Key Laboratory of ASIC and System, School of Microelectronics, Fudan University, Shanghai, 200433 China

**Keywords:** Band alignments, HfAlO dielectric, InP, Atomic layer deposition

## Abstract

X-ray photoelectron spectroscopy and high-resolution transmission electron microscopy have been used to determine interfacial properties of HfO_2_ and HfAlO gate dielectrics grown on InP by atomic layer deposition. An undesirable interfacial InP_x_O_y_ layer is easily formed at the HfO_2_/InP interface, which can severely degrade the electrical performance. However, an abrupt interface can be achieved when the growth of the HfAlO dielectric on InP starts with an ultrathin Al_2_O_3_ layer. The valence and conduction band offsets for HfAlO/InP heterojunctions have been determined to be 1.87 ± 0.1 and 2.83 ± 0.1 eV, respectively. These advantages make HfAlO a potential dielectric for InP MOSFETs.

## Background

As silicon-based complementary metal-oxide-semiconductor (CMOS) devices approach their fundamental limits when scaled down, the adoption of new technologies is urgently required to meet the demands for higher performance and less power dissipation integrated circuits. The integration of III–V compound semiconductors with high-*k* gate dielectrics is the leading candidate to address many of these issues [1–6]. III–V compound semiconductors including GaAs, InGaAs, and InP have drawn quite a lot of attention as alternative channel materials due to their high electron mobility and low effective mass over Si. Of these candidates, InP could be promising because it is a material with weak Fermi-level pinning effect and has a high electron saturation velocity (2.5 × 10^7^ cm/s) [7]. However, there are still some bottlenecks impeding the actual implementation of InP channel material. One of the major challenges is finding thermodynamically stable dielectric on InP surface with good interfacial properties like SiO_2_/Si counterpart [8]. Various chemical treatments have been extensively explored to achieve effective passivation on the InP surface. Such methods include NH_4_OH, (NH_4_)_2_S, H_2_S, and F treatment [7, 9–11].

Band alignment of high-*k*/InP interface is of great importance for InP-based MOSFET researches. Recently, Chou et al. measured the band offset between InP (100) and ALD Al_2_O_3_ with various passivation methods using internal photoemission (IPE) [12]. The barrier heights from the top of InP valence band (VB) to the bottom of Al_2_O_3_ conduction band (CB) and conduction band offset were determined to be 4.05 and 2.7 ± 0.10 eV, respectively. With a high *k* of ~20–25, HfO_2_ has been extensively studied as an alternative high-*k* gate dielectric both in Si-based and III–V technology. The barrier height at HfO_2_/InP interface is also measured using IPE by Xu et al. to be 3.89 eV for the sample without passivation treatment [13]. To improve the interfacial and thermal properties, gate dielectric engineering has been successfully adopted in high-*k*/III–V compound semiconductor technology by using a combination of Al_2_O_3_ and HfO_2_ films recently. Kim et al. reported that the In out-diffusion and the subsequent In-related phase generation can be effectively suppressed by introducing Al_2_O_3_ to the HfO_2_ film grown on InP. Moreover, intermixing of Al_2_O_3_ with HfO_2_ to form HfAlO on InP is expected to adjust the band offset. In this study, HfAlO gate dielectrics were grown on InP substrates using alternative cycles atomic layer deposition (ALD) of Al_2_O_3_ and HfO_2_ starting with an ultrathin Al_2_O layer. ALD has manifested itself as a technique suitable for the semiconductor industry due to its capability of growing uniform and conformal thin films [14]. Accurate controls over chemical stoichiometry and thickness at atomic scale can also be achieved by ALD. The ultrathin Al_2_O_3_ layer was introduced to the interface between HfAlO dielectrics and InP substrate to diminish the undesirable interfacial InP_x_O_y_ layer. The band offset and interfacial properties of the formed HfAlO gate dielectric grown on InP has been investigated using x-ray photoelectron spectroscopy (XPS). The energy band diagrams of the HfAlO/InP heterojunction is then constructed, which can provide vital information on fabricating InP MOSFET.

## Methods

The heterostructures were prepared on *n*-type (100) InP wafers with a doping concentration of ~1 × 10^17^ cm^−3^. Prior to the ALD process, the InP substrates were degreased using acetone and methanol for 5 min each, followed by a diluted 2% hydrofluoric acid (HF) solution etching for 1 min to remove native oxide. After cleaning, the substrate was immediately transferred to a BENEQ TFS-200 ALD reactor where HfO_2_ and HfAlO thin films were prepared at 300 °C. Thin HfAlO film was grown using alternative cycles ALD of Al_2_O_3_ and HfO_2_ starting with Al_2_O_3_. The precursors used were trimethylaluminum (TMA) and H_2_O for Al_2_O_3_ and tetrakis(ethylmethylamido)hafnium (TEMAH) and H_2_O for HfO_2_. Four sets of samples were prepared for XPS measurements: (1) a 4 nm thick HfO_2_ film grown on InP substrate to detect the interface property of the HfO_2_/InP heterojunction; (2) a 30 nm thick HfAlO film grown on InP substrate to measure the valence band maximum (VBM) and Al 2*p*_3/2_ of bulk HfAlO; (3) a 4 nm thick HfAlO film grown on InP substrate to determine the energy difference between Al 2*p*_3/2_ and In 3*d*_5/2_ at the HfAlO/InP heterojunction’s interface; and (4) a clean InP substrate (HF-dipped) to measure the VBM and In 3*d*_5/2_ of bulk InP.

High-resolution transmission electron microscopy (HR-TEM) was used to obtain the images of high-*k*/InP interface at atomic scale. Both TEM with low and high magnification were performed to investigate the interfacial profile and fringe atom arrangement. Accurate film thicknesses can also be measured directly. The XPS spectra were recorded using a Thermo Scientific ESCALAB 250 XPS system equipped with a monochromatic Al *Kα* source (*hv* = 1486.6 eV). The source power is 150 W (15 kV × 10 mA) at a takeoff angle of 90°. Scans with a step of 0.05 eV and pass energy of 20 eV were performed for 20 times for binding energy of specific elements. The chemical compositions for the prepared HfAlO thin film were detected to be 14.23% for Hf, 22.36% for Al, and 62.55% for O, respectively. Valence band scans with a step of 0.01 eV and pass energy of 20 eV were performed for 30 times for valence band spectra. Charge correction was performed using the known position of C 1*s* spectra at 284.8 eV. The XPS spectrometer energy scale was also calibrated using Cu 2*p*_3/2_, Ag 3*d*_5/2_, and Au 4*f*_7/2_ photoelectron lines located at 932.67, 368.26, and 83.98 eV, respectively.

## Results and Discussion

**Fig. 1:**
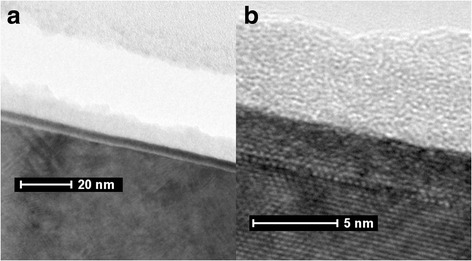
HR-TEM images of as-grown HfO_2_ thin films on InP substrate, with **a** low magnification and **b** high resolution

Figure 1 gives the TEM images of the as-deposited HfO_2_ film grown on InP. As shown in Fig. 1(a), the dielectric film in amorphous structure is observed in a large area with low magnification. Moreover, it can be seen clearly from the Fig. 1b, by high magnification, that an interlayer (~1 nm) exists at the interface between the HfO_2_ film and the InP substrate. The visible interlayer is inferred to consist of In-O and P-O compounds which can be assigned to the diffusion of In and P atoms into the HfO_2_ film.

**Fig. 2:**
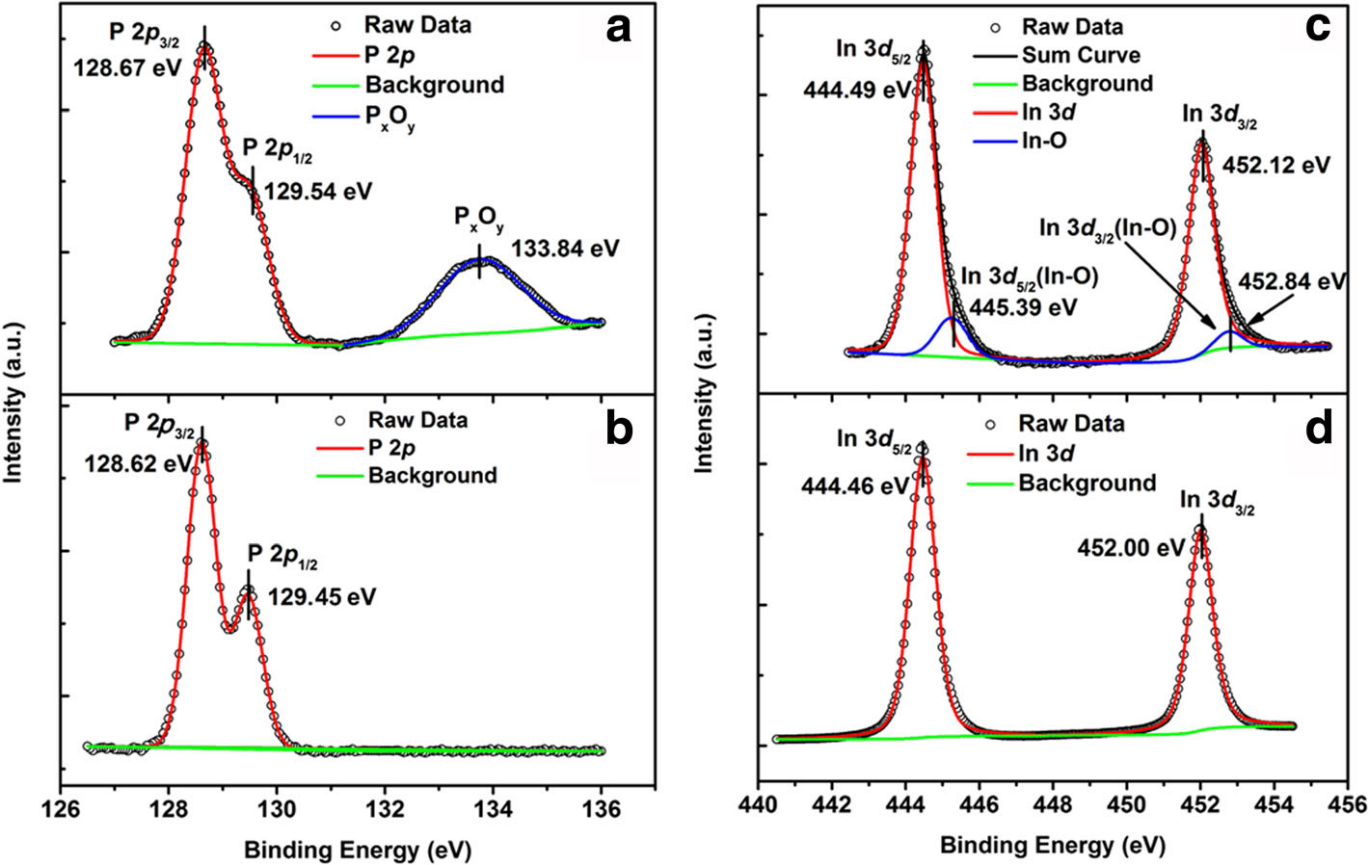
P 2*p* spectra for **a** HfO_2_ (4 nm)/InP heterojunction and **b** bulk InP; In 3*d* spectra for **c** HfO_2_ (4 nm)/InP heterojunction and **d** bulk InP

To further investigate the effect of HfO_2_ film deposition on the interfacial properties and the components of the interlayer, In 3*d* and P 2*p* XPS spectra for sample (1) (4 nm thick HfO_2_ sample on InP substrate) and sample (4) (bulk InP sample) are investigated and presented in Fig. 2. According to Fig. 2a, the P 2*p* core level spectrum attributed to the In-P bond from the substrate is composed of two peaks, P 2*p*_3/2_ (128.67 eV) and P 2*p*_1/2_ (129.54 eV), with a spin orbit split (SOS) of 0.87 eV. Compared with the P 2*p* XPS spectra of bulk InP sample, shown in Fig. 2b, an increase of the full width at half maximum (FWHM) of the P 2*p* for HfO_2_/InP sample was observed in the spectra range of 127.2–130.6 eV. This result is in agreement with the findings obtained by Dong et al. [15, 16]. They suggested that there is an increase in the distribution of the chemical bonding environments at the InP/HfO_2_ interface during the ALD process. It is ascribed to the increased surface disorder introduced by the reaction between TEMAH and the InP substrate. Moreover, a new peak for 4-nm-thick HfO_2_/InP is clearly observed at a binding energy of 133.84 eV. Investigation by Lu et al. shows the peak is assigned to the P-O bonding [11]. Besides, according to Fig. 2(d), the In 3*d* core level spectrum attributed to the In-P bond from the InP substrate is composed of two spin-orbit split peaks, In 3*d*_5/2_ (444.46 eV) and In 3*d*_3/2_ (452.00 eV), with a SOS of 7.54 eV [17]. However, as shown in Fig. 2(c), new peaks located at 445.39 and 452.84 eV are also found in the In 3*d* spectrum for HfO_2_ (4 nm)/InP sample, which are attributed to In-O bonding and cannot be detected in bulk InP. This spectrum further confirms the formation of InP_x_O_y_ layer at the interface of HfO_2_/InP heterojunction. According to investigations by Chen et al. and Driad et al., respectively*,* the high interface state density will result in a large frequency dispersion, a low breakdown voltage and a large gate leakage current for InP metal-oxide-semiconductor capacitors with HfO_2_ deposited directly on InP substrates [18, 19]. What is more, when the HfO_2_/InP heterojunction is fabricated into MOSFETs, the interlayer will cause a low transconductance and a large subthreshold swing.

**Fig. 3:**
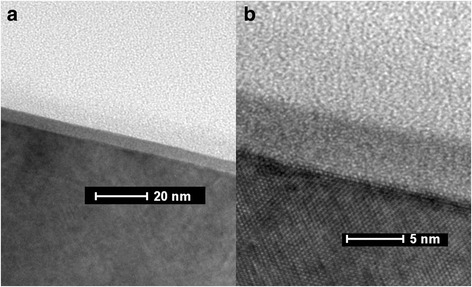
HR-TEM images of as-grown HfAlO thin films on InP substrate, with **a** low magnification and **b** high resolution

**Fig. 4:**
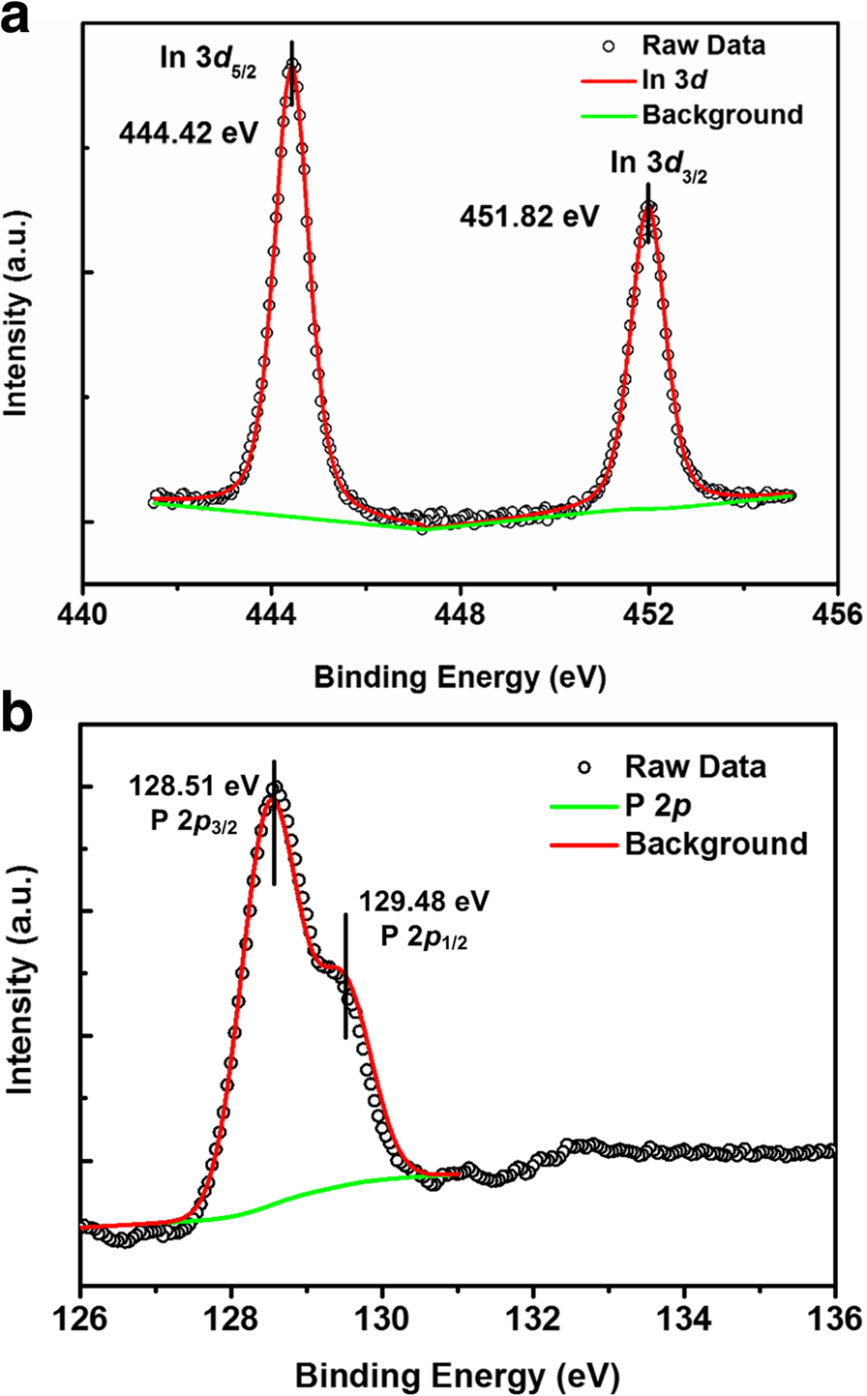
XPS narrow scans of **a** In 3*d* and **b** P 2*p* for HfAlO (4 nm)/InP heterojunction

An interfacial layer is easily formed as HfO_2_ deposited directly on InP substrate. The HfAlO layer is deposited on InP started with an ultrathin Al_2_O_3_ layer. Figure 3 shows the HR-TEM images with low and high magnification for the HfAlO (4 nm)/InP sample, which reveal an abrupt interface between HfAlO and InP. Chemical composition analysis for the HfAlO (4 nm)/InP sample was revealed by XPS as well, as shown in Fig. 4. Compared with the In 3*d* and P 2*p* spectra from the 4-nm-thick HfO_2_ grown on InP substrate, it is clear that In-O or P-O component completely vanish. The formation of a clean interface strongly indicates the effect of “self-cleaning,” and similar effect of thin Al_2_O_3_ film grown on GaAs by ALD has been studied by Hinkle et al. [20]. The results imply that a thin Al_2_O_3_ layer can passivate the InP surface by acting as a barrier layer to prevent the diffusion of In and P from substrate into the dielectric film. Similarly, the shape of P 2*p* spectrum from the InP bulk peak is different with the one from the pure InP substrate. It is indicated that the FWHM of the sample also increase due to the reaction between TMA and InP substrate during the ALD process.

The band alignment for HfAlO/InP heterostructure at the interface is then investigated, which is a crucial standard to measure the ability to suppress leakage current for devices. To determine the valence band offset (Δ*E*_v_) of HfAlO/InP, Kraut’s method is employed, which is based on the assumption that the energy separation between the valence band edge and core level (CL) of the substrate remains the same before/after dielectric deposition [21–23]. In this work, In 3*d*_5/2_, Al 2*p*_3/2_, and Hf 4*f*_7/2_ are selected as reference CLs for the substrate and high-*k* dielectrics, respectively. The CL spectra were fitted by Shirley background and a nonlinear Gaussian-Lorentzian line shape with a fixed spin orbit splitting to determine the respective CL positions. The Δ*E*_v_ value could be extracted from the following equations,

1 $$ \Delta {E}_{\mathrm{V}}^{\mathrm{HfAlO}/\mathrm{InP}} = {\left({E}_{\mathrm{In}\ 3{d}_{5/2}}-{E}_{\mathrm{V}\mathrm{BM}}\right)}_{\mathrm{bulk}\kern0.2em \mathrm{InP}}-{\left({E}_{\mathrm{CL}\kern0.2em \mathrm{peak}}-{E}_{\mathrm{V}\mathrm{BM}}\right)}_{\mathrm{bulk}\kern0.2em \mathrm{HfAlO}}-\Delta {E}_{\mathrm{CL}} $$

where Δ*E*_CL_ is the energy difference between In 3*d*_5/2_ CL spectrum and Al 2*p*_3/2_ CL spectrum at the interface of HfAlO (4 nm)/InP heterojunction. All of the spectra for thin high-*k* oxide/InP samples are referenced to the In 3*d*_5/2_ peak from clean InP sample to compensate for charging effects. The parameters extracted via XPS, for the samples studied, are listed in Table 1 for clarity.

**Table 1:** Binding energies (in eV) of core level spectra for all the samples and the valence band maximum (VBM) values (in eV) for the bulk InP and HfAlO (30 nm)/InP samples

	InP (bulk)	HfAlO (30 nm)/InP	HfAlO (4 nm)/InP
In 3*d*_5/2_	444.46	–	444.42
Hf 4*f*_7/2_	–	17.61	17.32
Al 2*p*_3/2_	–	74.46	74.21
VBM	0.79 ± 0.05	2.89 ± 0.05	–

The errors in the peak positions and the VBM values are ±0.05 eV

**Fig. 5:**
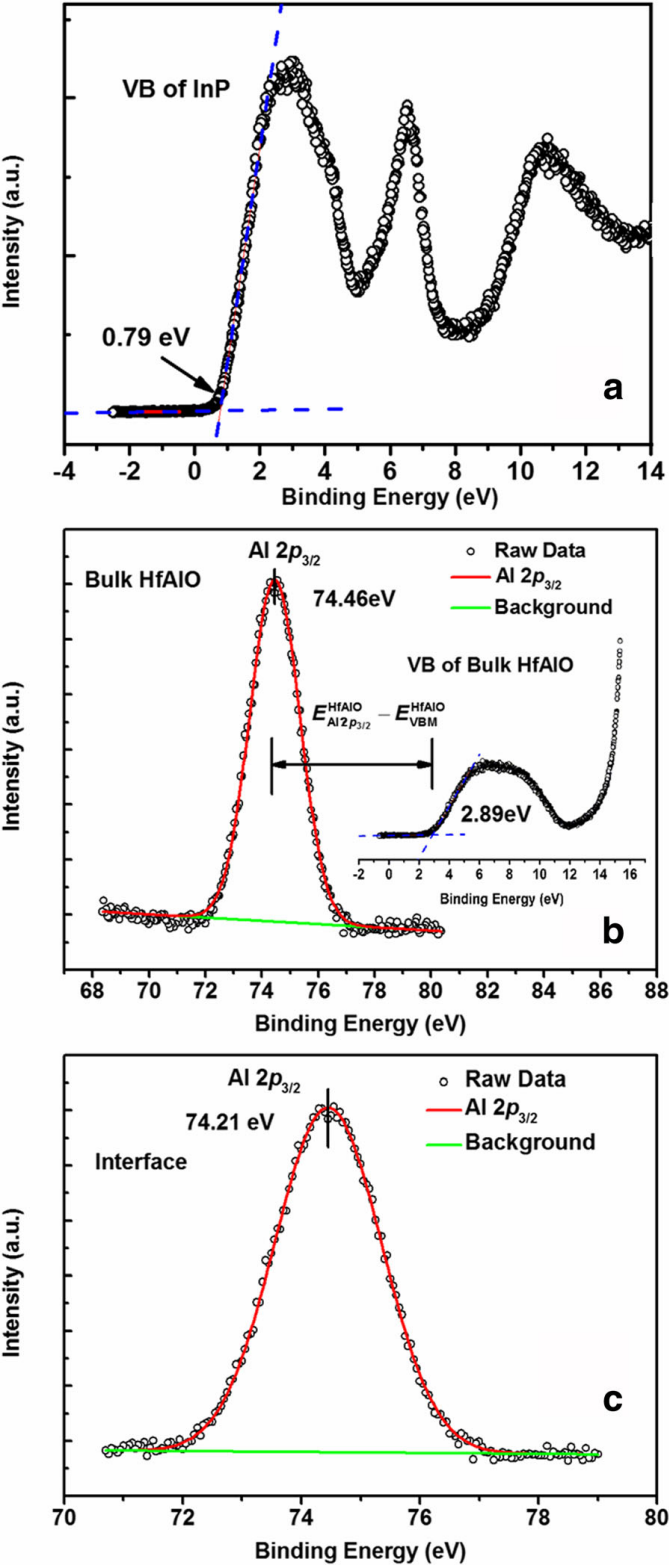
**a** Valence band (VB) spectrum of bulk InP. **b** XPS narrow scan of Al 2*p*_3/2_ and VB spectrum in bulk HfAlO (30 nm thick). **c** XPS narrow scan of Al 2*p*_3/2_ in the HfAlO (4 nm)/InP interface

Figure 5 shows VB spectra of InP substrate and 30-nm-thick HfAlO sample as well as and the Al 2*p*_3/2_ spectra of HfAlO films. The VBM positions were determined by linear extrapolation of the leading edges of the VB spectra recorded from the InP substrate and the bulk HfAlO films to the base lines in order to account for the tail induced by instrument resolution. The uncertainty of the VBM positions should be lower than 0.05 eV because considerable accordance of the fitted lines to the measured data has been obtained. Through extrapolating a linear fitting of the leading edges of VB spectra to the base lines, the VBM position is determined to be 0.79 ± 0.05 eV and 2.89 ± 0.05 eV for InP substrate and 30-nm-thick HfAlO films, respectively. The VBM value of bulk HfAlO sample is in good agreement with the previously reported one by Yu et al. [24]. Figure 5b depicts a single and symmetrical peak located at 74.46 eV in the CL spectrum of Al 2*p*_3/2_, indicating the uniform Al-O bonding state in the 30-nm HfAlO film. Furthermore, the CL spectrum of Al 2*p*_3/2_ for HfAlO (4 nm)/InP heterojunction is shown in Fig. 5c. Compared with thick HfAlO (30 nm)/InP sample, Al 2*p*_3/2_ peak shifts to 74.21 eV at the interface. With all the obtained values above, ${\left({E}_{\mathrm{In}\ 3{d}_{5/2}}-{E}_{\mathrm{VBM}}\right)}_{\mathrm{In}\mathrm{P}}$, ${\left({E}_{\mathrm{Al}\ 2{p}_{3/2}}-{E}_{\mathrm{VBM}}\right)}_{\mathrm{HfAlO}}$, and Δ*E*_CL_ are calculated to be 443.67 ± 0.05, 71.57 ± 0.05, and 370.21 eV, respectively. As a result, the Δ*E*_v_ value of HfO_2_/InP heterojunction is determined to be 1.89 ± 0.1 eV using Eq. (1). To improve the accuracy of the VBO value determined by XPS, the combination of Hf 4*f* and In 3*d* is also used to obtain the VBO of HfAlO/InP interface. Relative data is summarized in Table 1, and the VBO is determined to be 1.85 ± 0.1 eV. To reduce the measurement error, the VBO value of HfAlO/InP interface is then calibrated to be 1.87 ± 0.1 eV by averaging the two VBO values.

The conduction band offset (Δ*E*_c_) at the high-*k*/InP interface can be determined by the following equation:

2 $$ \Delta {E}_{\mathrm{c}}={E}_{\mathrm{g}}^{\mathrm{high}\hbox{-} k}-{E}_{\mathrm{g}}^{\mathrm{InP}}-\Delta {E}_{\mathrm{v}} $$

The energy band gaps (*E*_g_) measured by spectroscopic ellipsometry are 5.70 and 6.04 eV for as-deposited HfO_2_ and HfAlO films, respectively. Using 1.34 eV energy gap for InP [25], the Δ*E*_c_ at the HfAlO /InP interface is thus calculated to be 2.83 ± 0.1 eV. According to the investigation above, both of the Δ*E*_v_ and Δ*E*_c_ values are larger than 1 eV, which means the HfAlO film supplies enough barrier heights for both electrons and holes. Compared with the Δ*E*_c_ extracted to be 2.55 eV at the interface of HfO_2_/InP by Xu et al. [13], the Δ*E*_c_ for HfAlO/InP is a little larger. Furthermore, the Δ*E*_v_ at the interface of HfO_2_/InP can also be calculated to be 1.81 eV using equation (2), which is a little smaller than that of HfAlO/InP heterojunction. As a result, the dielectric HfAlO film can suppress both the electrons induced and holes induced leakage current more effectively than the HfO_2_ film when InP is chosen as channel material. Considering the better interface condition and higher potential barrier heights, HfAlO can be relatively preferable than HfO_2_ as the dielectric film for InP.

## Conclusions

In summary, interfacial properties for HfO_2_/InP and HfAlO/InP heterojunctions have been studied by XPS and HR-TEM. When HfO_2_ is deposited on InP substrate by ALD, an undesirable interfacial InP_x_O_y_ layer is easily formed at the HfO_2_/InP interface, which will degrade the electrical properties. Fortunately, a thin Al_2_O_3_ layer can act as a barrier layer to form an abrupt interface between HfAlO and InP channel layer. The band alignment at the interface of HfAlO/InP heterojunction was studied, and the VBM for HfAlO/InP is 1.87 ± 0.1 eV at interface, leading to a Δ*E*_c_ of 2.83 ± 0.1 eV for HfAlO/InP based on the analysis. This result indicates that HfAlO dielectric film can supply sufficient barrier heights for holes and electrons when InP is chosen as channel material. More researches are needed to make further efforts to optimize the heterojunction’s interface condition.
